# Exploring operational boundaries for acoustic concentration of cell suspensions

**DOI:** 10.1007/s00253-024-13215-1

**Published:** 2024-06-19

**Authors:** Amaury de Hemptinne, Pierre Gelin, Ilyesse Bihi, Romain Kinet, Benoit Thienpont, Wim De Malsche

**Affiliations:** 1 Department of Chemical Engineering, µFlow Group, Vrije Universiteit Brussel, 1050 Brussels, Belgium; 2 GSK, Rixensart, Belgium

**Keywords:** Acoustophoresis, Cell concentration, Cavitation, Contrast factor

## Abstract

**Abstract:**

The development of a standardized, generic method for concentrating suspensions in continuous flow is challenging. In this study, we developed and tested a device capable of concentrating suspensions with an already high cell concentration to meet diverse industrial requirements. To address typical multitasking needs, we concentrated suspensions with high solid content under a variety of conditions. Cells from *Saccharomyces cerevisiae*, *Escherichia coli*, and Chinese hamster ovary cells were effectively focused in the center of the main channel of a microfluidic device using acoustophoresis. The main channel bifurcates into three outlets, allowing cells to exit through the central outlet, while the liquid evenly exits through all outlets. Consequently, the treatment separates cells from two-thirds of the surrounding liquid. We investigated the complex interactions between parameters. Increasing the channel depth results in a decrease in process efficiency, attributed to a decline in acoustic energy density. The study also revealed that different cell strains exhibit distinct acoustic contrast factors, originating from differences in dimensions, compressibility, and density values. Finally, a combination of high solid content and flow rate leads to an increase in diffusion through a phenomenon known as shear-induced diffusion.

**Key points:**

• *Acoustic focusing in a microchannel was used to concentrate cell suspensions*

• *The parameters influencing focusing at high concentrations were studied*

• *Three different cell strains were successfully concentrated*

**Graphical abstract:**

**Figure:**
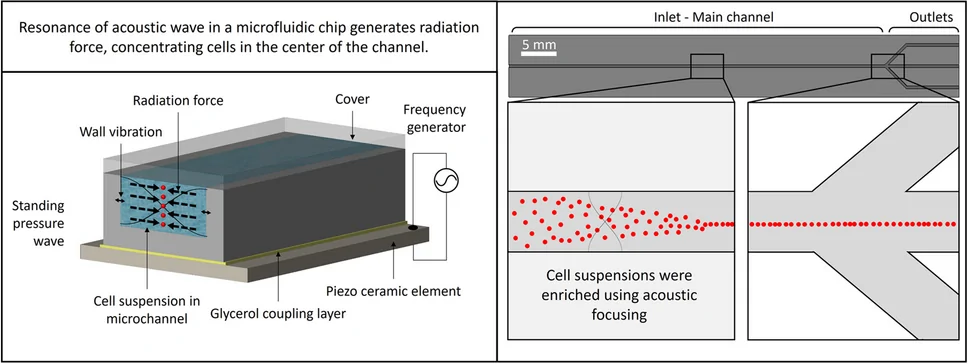

**Supplementary Information:**

The online version contains supplementary material available at 10.1007/s00253-024-13215-1.

## Introduction

The isolation and enrichment of particles and cells are key techniques used in various fields. The increasing demand for cell separation in multiple disciplinary research fields is well observed in engineering and regenerative medicine, but also in many other areas such as biochemistry, electrical engineering, physics, and materials science (Tomlinson et al. 2013). On the one hand, isolation involves creating a selective barrier that allows one component to pass through freely while retaining or deflecting other components (Xie et al. 2020). Usually, this is the initial step where the target particles and cells are separated from the rest of the mixture. Various methods like centrifugation, filtration, magnetic separation, and immunoprecipitation can be used to achieve this goal (Warrick et al. 2010). On the other hand, enrichment involves increasing the concentration of the desired cells or particles in the sample (Xie et al. 2020). Usually, this step comes after isolation, where the concentration is relatively low, especially if the target particles and cells are rare or present in small amounts.

Enrichment is challenging when dealing with living cells in industrial manufacturing. Moreover, larger quantities are needed for production purposes. Additionally, concentration can greatly facilitate detection, improving the overall sensitivity and accuracy of assays (Warrick et al. 2010). In many studies, isolation and enrichment techniques overlap (Xie et al. 2020). Centrifugation, for instance, can both separate and enrich particles or cells based on their densities. By adjusting the centrifugation conditions, either heavier particles/cells can be concentrated by removing the supernatant or lighter particles can be separated from the heavier ones without significant enrichment. To achieve enrichment of the lighter particles, a second centrifugation step with distinct conditions is performed after the initial separation (Xie et al. 2020).

In recent years, microfluidics has shown remarkable success in isolating and enriching various particles, including cells with diverse characteristics. Different microfluidic methods, such as acoustics, optics, dielectrophoresis, and filtration, have been proven to be effective for these tasks (Xie et al. 2020). However, limited attention has been given to handling highly concentrated samples due to sensitivity to clogging of methods like microfluidic filtration or DLD (deterministic lateral displacement) (Xie et al. 2020). Optofluidics, while offering high resolution, suffers from low throughput (Wu et al. 2016). Dielectrophoresis leads to Joule heating and requires dedicated medium, which can be detrimental to cells. Xie et al. (2020) presented a review of the different microfluidic methods for isolation and enrichment with their advantages.

**Fig. 1:**
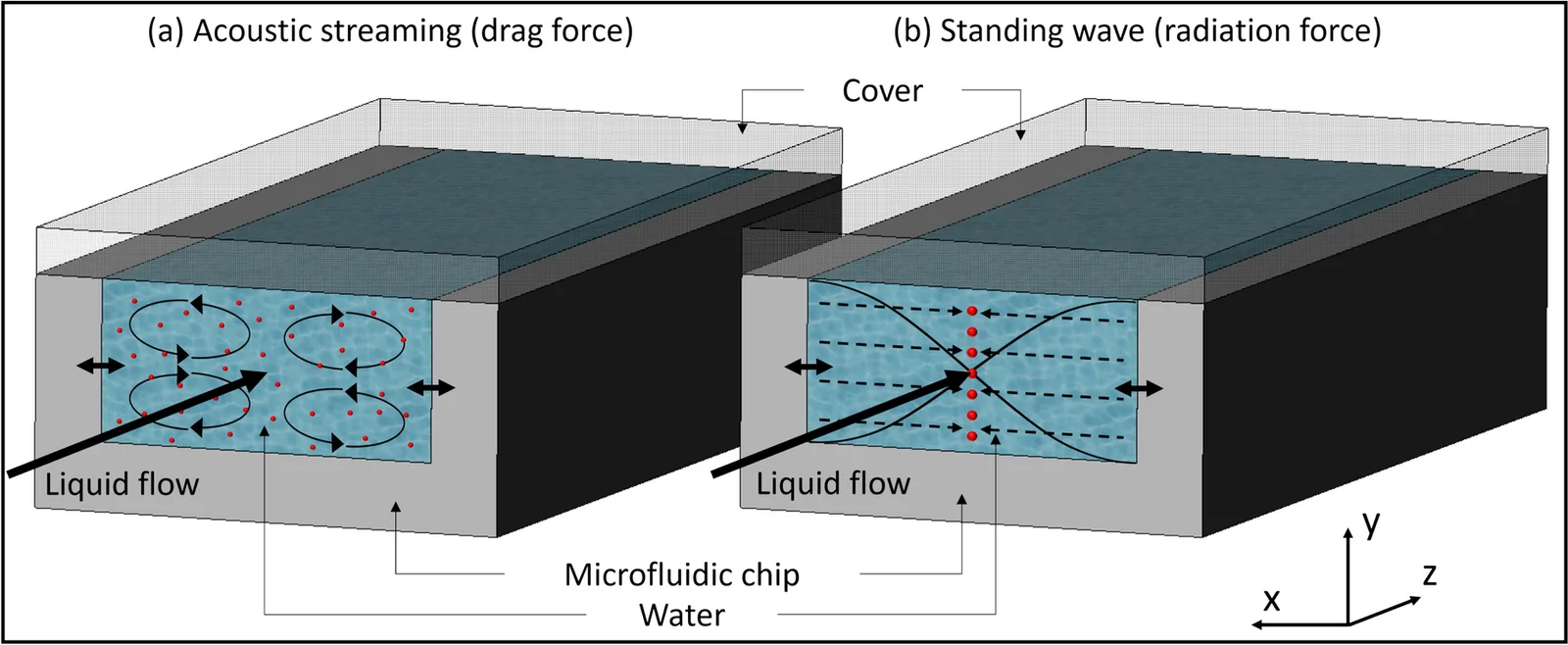
Visualization of the two effects resulting from a standing bulk acoustic wave (BAW) with a single node in a rectangular microfluidic channel. **a** Liquid streams are depicted on the left and, **b** on the right, the dashed lines arrows indicate the radiation force arising from the scattering of acoustic standing waves pressure waves represented by the sinusoidal shape lines

Consequently, acoustics appears to be the method of choice for handling high concentrations during the enrichment process, offering promising advantages in this regard. Acoustofluidics can be operated in two main modes (Lenshof et al. 2012; Leibacher et al. 2015). In the surface acoustic wave (SAW) mode, particles and cells are manipulated by acoustic waves generated by an interdigitated transducer attached to one of the surfaces of the device. The wave propagates then along this surface. The second mode is bulk acoustic wave (BAW) mode. Ultrasonic standing waves are generated by a bulk piezoelectric transducer (PZT) placed in intimate contact with the device. Standing waves are transmitted in a liquid-filled channel and resonance is observed in an acoustically hard material, often silicon (Leibacher et al. 2015). Standing BAWs in a rectangular channel produce two effects that influence the position of suspended cells (Bruus 2008; Wiklund et al. 2012; Ley and Bruus 2016; Gelin et al. 2021). On the one hand, liquid streaming takes place, hence inducing a drag force on the cells (Fig. 1a). On the other hand, scattering of acoustic pressure waves in the liquid produces a radiation force along the width of the channel, leading to focusing the particles or cells (Ley and Bruus 2016; de Hemptinne et al. 2023a) (Fig. 1b). In a standing acoustic wave, the pressure remains constant at the nodes. Elsewhere, the pressure varies as a function of time, the largest values are obtained at the anti-nodes. Acoustic focusing is a non-invasive method that has been reported not to harm living cells under common operational conditions (Wiklund 2012). Based on the applied frequency, one or several nodes can be generated in the channel. The streaming related vortex flows and the pressure waves with a single node are respectively depicted in Fig. 1 a and b.

The radiation force, ${F}_{r}(x)$, exerted on the suspended particles can be expressed as follows:

1 $${F}_{r}\left(x\right)=n\;4\;{\pi }^{2}\phi\;\left(\widetilde{\rho\;},\widetilde{\kappa\;},\widetilde{\delta\;}\right)\frac{{r}_{p}^{3}{E}_{\mathrm{ac}}}{w}\mathrm{sin}(n2\pi \frac{x}{w}+n\pi )$$

with n the number of nodes, ${r}_{p}$ the particle/cell radius, ${E}_{\mathrm{ac}}$ the acoustic energy density, w the channel width and x the position along the channel width (Barnkob et al. 2012), and f is the frequency of the acoustic wave. The acoustic contrast factor ϕ depends on three parameters. $\widetilde{\rho }$ and $\widetilde{\kappa }$ are respectively the ratio of density and compressibility between the liquid and the particle and $\widetilde{\delta }$ is the ratio between the acoustic boundary layer thickness and the particle radius (Bruus 2008; Wiklund 2012; Ley and Bruus 2016; Gelin et al. 2021). The thickness of this boundary layer depends on the liquid viscosity and the frequency applied on the transducer. The sign of the contrast factor indicates the direction of the radiation force. A higher density and lower compressibility of the particle than that of the liquid results in a radiation force that is oriented in the direction of the node. More details about the theoretical model and equations are given in literature (Barnkob et al. 2012; Muller et al. 2012).

In microfluidics, the small size of the channels generally results in a low Reynold number (Re<1). The drag force can in this case be described by the Stokes law (Stokes 1851).

2 $${F}_{d}\left(x,y\right)=6\pi \mu {r}_{p}v$$

with μ the liquid dynamic viscosity and v the liquid velocity. To understand the particle behavior, Newton’s second law can be applied. Equation 3 describes the forces acting on particles in stop flow condition. Following previous studies, in similar conditions, the time scale of the acceleration is much smaller than that of the translation (Reddy et al. 1996; de Hemptinne et al. 2022, 2023b). It allows neglecting inertial effects, i.e., the sum of the forces is zero.

3 $${F}_{r}+{F}_{d}=0$$

As stated in Eqs. 1 and 2, the radiation force is proportional to the cube of the particle radius (${F}_{r}\propto {r}_{p}^{3}$) and the Stokes drag force is directly proportional to the particle radius (${F}_{d}\propto {r}_{p}$). Consequently, at constant streaming flow, large particles will mainly be affected by the radiation force while small particles will follow the flow under the effect of the drag force (Barnkob et al. 2012).

When both forces have an equal value, it results in zero velocity. In this condition, a critical particle diameter ${d}_{c}$ can be determined. With a single node, for a polystyrene particle in water in a rectangular channel that is 377 µm wide, a ${d}_{c}$ of 1.4 µm was calculated (Barnkob et al. 2012).

Below the critical diameter, cells flow along with vortices associated to acoustic streaming. When the cell size is larger than ${d}_{c}$, focusing takes place near the node(s), i.e., in the center of the channel for a single node. Typical cell sizes vary between 0.1 and 100 µm, for a given cell type the size also depends on the stage of development (Willey et al. 2017). Both streaming, below ${d}_{c}$, and radiation forces, above ${d}_{c}$, can therefore be exploited to take advantage of BAWs in the field of microbiology (Spengler et al. 2003; Spengler and Coakley 2003; Barnkob et al. 2012; Muller et al. 2012).

As mentioned earlier, the radiation force can be exploited for separation and enrichment purposes. Petersson et al. (2004) set up a continuous separation device resembling the one utilized in this study for the purpose of segregating various kinds of particles. The apparatus consists of a single inlet and three outlets, with the middle exit being designated for collecting dense and less compressible particles, while the other two exits are used for the remaining particles. The separation channel was evaluated in vitro, using polyamide spheres suspended in water, showing separation efficiencies approaching 100%. The system was also evaluated on whole blood to separate lipid particles and erythrocytes. More than 80% of the lipid particles could be removed while approximately 70% of the erythrocytes were collected in one third of the original fluid volume.

As indicated in Table 1, Carugo et al. (2014) developed a device to enrich a *E. coli* suspension with a concentration of 10^4^ cells/ml. Bacteria were levitated toward a glass surface under the effect of acoustic radiation forces. Multiple outlets (2 or 3) were used to enrich the cell suspension by removal of clarified liquid. Using the developed device, a significant increase in bacterial concentration has been achieved, up to a factor of 14.3.

**Table 1:** Studies reporting continuous-flow acoustic concentration of *Saccharomyces cerevisiae*, *Escherichia coli*, and CHO suspensions and corresponding concentration. The present work treats suspension of higher concentration for *E. coli* and *S. cerevisiae*. Concentration of CHO suspension is comparable

Author	Cell type	Initial concentration
Li et al. (2007) Present paper	*S. cerevisiae* *S. cerevisiae*	1.0 10^7^ 2.65 10^9^
Carugo et al. (2014) Present paper	*E. coli* *E. coli*	1.0 10^4^ 8.21 10^8^
Banerjee et al. (2021) Present paper	CHO CHO	2.5 10^7^ 2.25 10^7^

With alternative designs, Banerjee et al. (2021) worked on enrichment of a Chinese hamster ovary cell (CHO) suspension of 2.5 10^7^ cells/ml with acoustics and specifically studied the agglomeration phenomenon to predict cell size distribution. In their system, 90% of the cells were focused and recovered at the outlet of the chip. Li et al. (2007) concentrated a *Saccharomyces cerevisiae* (yeast) suspension of 10^7^ cells/ml using asymmetric surface acoustic wave propagation on a substrate upon which a droplet of the suspension is placed.

The physics of acoustophoresis in microchannels, in which diluted suspensions are subjected to acoustic standing waves, is well understood (Jönsson et al. 2004; Petersson et al. 2004, 2005; Bruus 2012; Karthick and Sen 2016). In these diluted suspensions, interactions between particles are however often neglected (Weiser et al. 1984). In the case of high solid load suspensions, this is no longer correct. Blood is a representative suspension with a high solid content which was already studied extensively in literature (Di Carlo et al. 2007; Lenshof et al. 2009; Karthick and Sen 2016; Gao et al. 2020). Lenshof et al. (2009) used acoustic focusing and a specific chip geometry to prepare diagnostic plasma from whole blood. The clarified liquid was in this study targeted. In order to handle high concentrations, the chip has a long channel with multiple outlets in the middle (bottom) of the channel to remove blood cells which are already focused. The progressive decrease of the concentration allowed to recover a purified plasma in a final structure with three outlets, the two outer outlets recovering the clarified plasma.

Some studies in literature have targeted enrichment of suspensions using acoustofluidics. They are however dedicated to study the influence of specific aspects of the process, as increasing solid content (Di Carlo et al. 2007; Lenshof et al. 2009; Karthick and Sen 2016, 2017; Gao et al. 2020), increasing flow rate (Wu et al. 2018; Hammarström et al. 2020), or cell strain comparison (Augustsson et al. 2010; Ai et al. 2013). A generic approach, facing these different aspects at the same time, is however required in order to develop an efficient tool that can be widely used.

In this work, three different cell strains, a yeast (*S. cerevisiae*), a bacterium (*E. coli*), and a mammalian cell line (CHO) were enriched in a BAW device. We explored the influence of and interplay between different key parameters. Different values of initial concentration, flow rate, channel depth, and applied potential on the PZT are explored.

## Materials and method

### Microfluidic chips

**Fig. 2:**
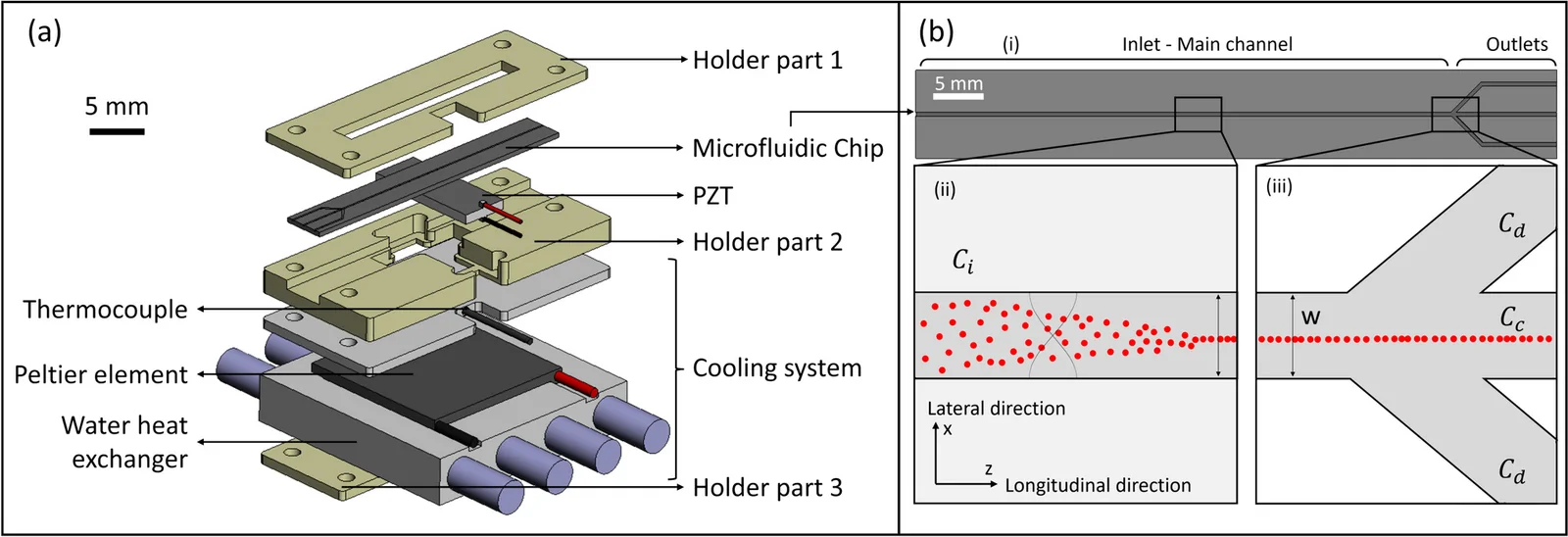
Illustration of the acoustofluidic setup and the chip geometry. **a** Acoustofluidic setup. From top to bottom, holder part 1, microfluidic chip, piezoelectric transducer (PZT), holder part 2, aluminum plate wearing the thermocouple, Peltier element, water heat exchanger, and holder part 3. **b** Chip geometry (i). The initial suspension was infused at the left-hand side, cells were subsequently focused in the center of the main channel by the radiation force (ii). Liquid left the chip through the three outlets while cells exit only through the central one (iii)

The microfluidic chip, illustrated in Fig. 2b, consists of an inlet and a main channel dividing in three outlets. Under the influence of the radiation force, cells are focused near the pressure node, in the center of the main channel, and leave the chip through the central outlet. The clarified liquid exits the chip through the two outer outlets.

The microfluidic chips have been fabricated in the MESA + cleanroom (UTwente, Netherlands) (Gelin et al. 2021; de Hemptinne et al. 2023a). The channels were patterned in a positive resist (Olin 907–12), with mid-UV lithography. In a second step, they were etched by Bosch-type deep reactive ion etching in a silicon wafer with a thickness of 525 µm (Adixen AMS100SE, Pfeiffer Vacuum SAS, Annecy, France). Next, the resist was removed with oxygen plasma and nitric acid. Next, the silicon wafer was sealed by anodic bonding with Pyrex wafers with a thickness of 500 µm and subsequently diced in individual chips.

The chips had a channel width (*w*) of 375 µm, with a theoretical resonance frequency at 2.00 MHz. The main channel was 5.3 cm long. Two different channels depths, 250 µm and 435 µm, were tested.

### Acoustofluidic setup

The chips were assembled into an acoustofluidic setup, illustrated in Fig. 2a. They were placed on a piezoelectric transducer (PZT) (15 × 20 × 1 mm, APC International Lt., Mackeyville, PA, USA), with a resonance frequency at 2.00 MHz, generating standing pressure waves in the main channel. A thin layer composed of 200 µL of glycerol facilitated coupling of the PZT with the chips. The PZT was controlled by a function generator (AFG1062, Tektronix UK Ltd., Bracknell, UK). The applied potential was amplified by a radio-frequency power amplifier (210L, Electronics & Innovation, Rochester, NY, USA) with a power output of 10 W (Gelin et al. 2021). A cooling system was placed below the PZT to control the temperature and prevent heating. The complete assembly was held together by a PMMA holder, in-house milled with a Computer Numerical Control (CNC) machine (Datron Neo, Datron, Mühltal, Germany). Holders were held with screws tightened with a torque of 15 cNm (de Hemptinne et al. 2023a).

The cells suspensions were infused in the chips with a pressure control unit (LineUP serie, Fluigent, Le Kremlin-Bicêtre, France). Capillaries were glued in the inlet and outlets with UV glue (Ormocore from microresist technology GmbH, Berlin, Germany). Capillaries (CM Scientific, Silsden, UK) had an inner and outer diameter of 75 µm and 200 µm, respectively for the chip with 250 µm deep channels and 200 µm and 350 µm respectively for the chip with 435-µm deep channels.

The cooling system was composed of three parts: an aluminum piece, in which a thermocouple was placed, a Peltier element (Peltier Module 88.2W, 9A, 15.8 V, 30 × 30 mm, European Thermodynamics Ltd, Kibworth, UK) and in conclusion a cooling aluminum piece, in which water circulated at 10 °C. Both aluminum pieces were milled with the CNC machine. The water was stored in a cooling bath (F250 Compact recirculating cooler, JULABO GmbH, Seelbach, Germany). A microcontroller (Arduino UNO R3, https://www.arduino.cc/) was used to control a feedback loop and a power supply (Stamos S-LS30, 0-30 V, 0-5A, RS PRO, Bremen, Germany) to adapt the potential applied to the Peltier element. More details on the cooling system are given in the results section.

### Particles and cell strains

Three cell strains, grown at GlaxoSmithKline (GSK, Rixensart, Belgium), were used: *S. cerevisiae* (yeast) (DC5), *E. coli* (BLR (DE3), a *recA*-deficient derivative of *E. coli* BL21(DE3)) and CHO.

The original suspensions concentrations were assessed at GSK using the plate count method (Willey et al. 2017; Beal et al. 2020), a common technique in biology, with the results expressed in colony-forming unit per ml (CFU/ml). In this method, the initial cell suspension is successively diluted. The final dilution is spread onto bacterial culture plates for incubation and colony counting. The number of CFU per mL is estimated by multiplying colony count by dilution multiple. This protocol has the advantage of being well established and insensitive to non-viable cells and debris. It however has the disadvantages of being labor-intensive (Beal et al. 2020). It also underestimates the real concentration due to merging colonies during the growing process. The actual concentrations of the suspensions are therefore potentially higher than the numbers presented here.

The concentrations of the different samples (before and after acoustic processing) were estimated with optical density measurements (Beal et al. 2020) in cells/ml. Calibration curves were established with successive dilutions of the original sample, hence used as a standard. The absorption was measured, at 670 nm, on samples of 150 µL, with a spectrophotometer (Synergy MX from BioTek company, Winooski, VT, USA).

In microfluidics, the concentration in particle suspension is often expressed in solid content, which is linked to the dimension of the particles:

4 $$\mathrm{SC}=100 \frac{{V}_{c}}{{V}_{c}+{V}_{l}}$$

with $\mathrm{SC}$ the solid content [%], ${V}_{c}$ the volume of a cell, and ${V}_{l}$ the volume of liquid. The solid content of the suspensions follows directly from the cells dimension, which is function of several parameters, such as age or division cycle (Trueba and Woldringh 1980). The different results in % v/v, based on the cells/ml values, are therefore to be used with caution.

The solid contents of the suspensions were calculated based on the concentration in CFU/ml and the theoretical cells dimensions. Yeast cells were considered with an ellipsoid shape (Dickinson and Schweizer 2017) of 9× 7 µm, with an initial concentration of 2.65 10^9^ CFU/ml. The calculation of the standard yeast suspension resulted in a solid content of 37.96% v/v. With their cylindrical shape, *E. coli* cells (Willey et al. 2017) are in average 4 µm long with a diameter of 1.3 µm. A concentration of 8.21 10^8^ CFU/ml corresponds to a solid content of 0.44% v/v. CHO cells have a typical dimension of 15 µm. The calculated solid content of the standard suspension, containing 2.25 10^7^ CFU/ml, was 3.82% v/v.

Fluorescent polystyrene particles with a diameter of 5.19 µm (PS-FluoGreen-5.0, microparticle GmbH, Berlin, Germany) were used to measure the radiation velocity. The displacement and the velocity of particles were analyzed with ImageJ (https://imagej.net/ij/).

Particles and cells were visualized with an inverted microscope (IX71 Olympus Corporation, Tokyo, Japan) equipped with a charged couple device camera (C9100-13, Hamamatsu Photonics, Hamamatsu, Japan). The yeast and CHO suspensions were observed in visible light. The fluorescent particles and *E. coli* suspension were observed in fluorescence mode.

During experiments, the cells were kept in suspension in the inlet reservoir with a magnetic stirrer to avoid their sedimentation.

Following each experiment, the chip underwent a cleaning process involving the infusion of various liquids. For experiments involving cells, a sequence of distilled water, urea, and distilled water was infused successively over a period of 5 min each. In experiments with PS particles, a sequential infusion of water, ethanol, ethyl acetate, ethanol, and water was performed with a duration of 2 min for each step.

## Results

### Temperature control

**Fig. 3:**
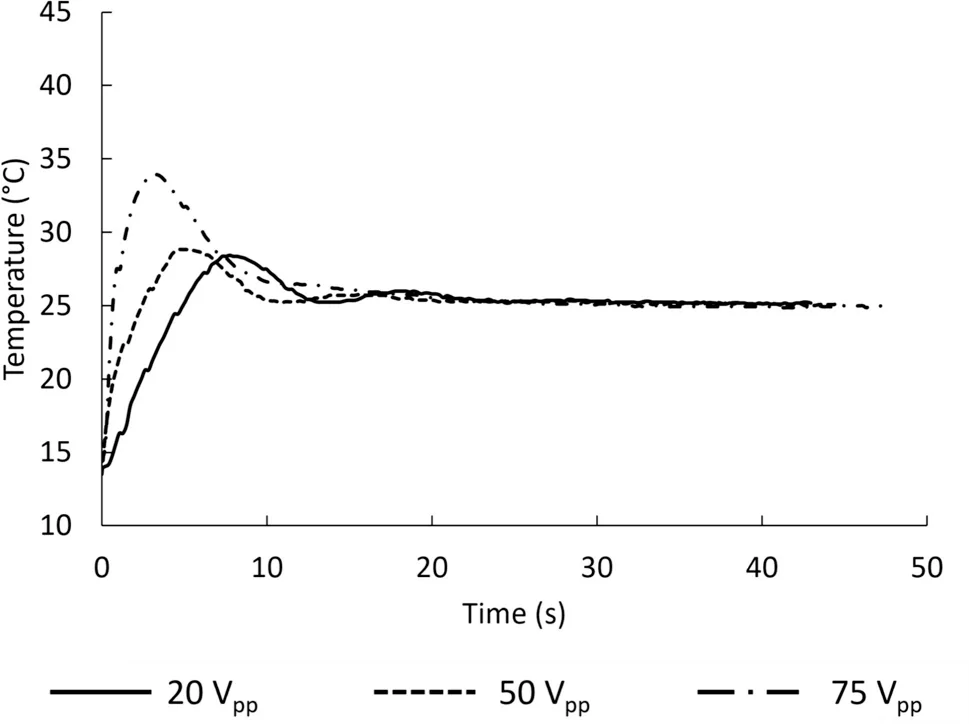
Thermocouple temperature readings as a function of the time with temperature controller when three different potentials were applied on the PZT. The temperature setpoint was 25 °C

Control of the chip temperature (Fig. 2a) was performed using a PID regulation (proportional, integral, derivation), optimized with the Ziegler-Nichols method (Ziegler et al. 1942). The thermocouple sent the temperature to a microcontroller, which in turn sent instructions to the laboratory power supply. Finally, the power supply drives the Peltier element to transfer heat. Figure 3 shows the temperature of the thermocouple as a function of time with cooling when applying 20, 50, and 75 *V*_pp_.

Without temperature control, at 20 *V*_pp_ heating remained limited but at 75 *V*_pp_, the temperature of the thermocouple reached 43 °C in 58 s. Clearly, in a biocompatible device, the effect must be considered and controlled. With the PID controller, with a setpoint of 25 °C, the temperature was rapidly stabilized, for instance in about 10 s when applying 75 *V*_pp_.

### Cell suspension concentration

The concentration of the suspensions was evaluated using absorbance at a wavelength of 670 nm. Samples from acoustic enrichment typically presented very high absorbance (in the non-linear absorbance region) due to high concentrations. Successive dilutions were therefore done before conducting absorbance measurements. The measured concentration value in cells/ml was finally multiplied by the dilution factor to recover the original concentration.

**Fig. 4:**
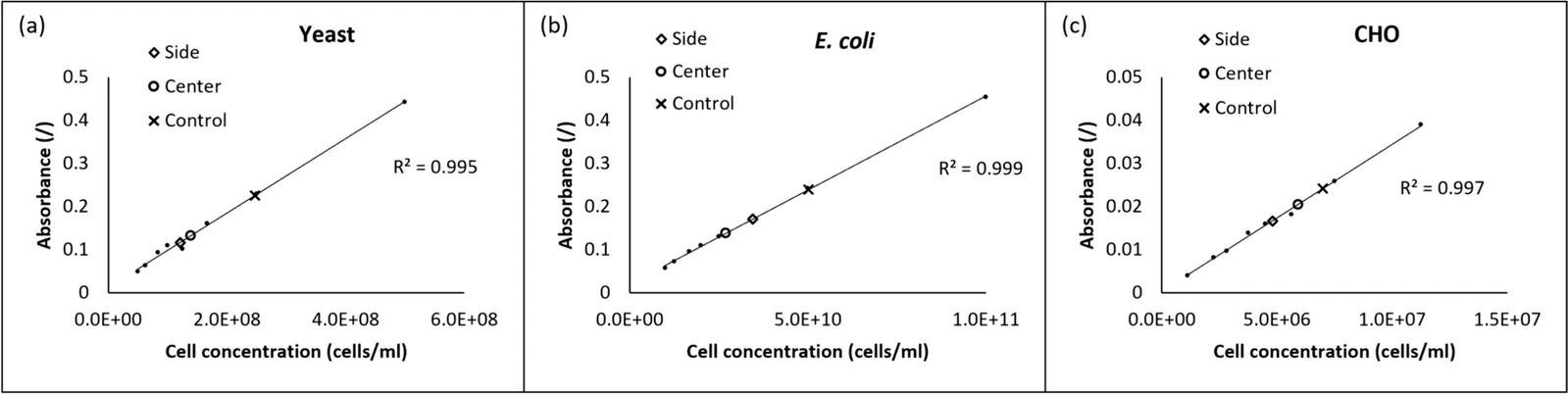
Calibration curves (dots lines) of the three cell strains, **a** yeast, **b** *E. coli*, **c** CHO. Each chart also contains the measurements of the samples with (side and center) and without (control) acoustic treatment. The samples analyzed in these charts correspond to the reference number Y2, E2, and C2 in Table 3 developed further

Figure 4 displays three charts, each corresponding to different strains, illustrating the calibration curves, the suspension without acoustic enrichment (control), the enriched suspensions (center channel), and the diluted suspensions (side channels).

Prior to conducting measurements for the calibration curve, initial suspensions of *E. coli* and yeast were first diluted to achieve an absorbance below 1. Subsequent to acoustofluidic treatment, additional dilutions were performed to ensure that the absorbance values aligned with the calibration curves. For instance, Table 2 illustrates the impact of these dilutions and acoustic treatment on the concentration of a yeast suspension with an initial concentration of 5.30 10^8^ cells/ml (sample Y2 in Table 3).

**Table 2:** The first column identifies the type of sample analyzed, the second column estimates the effect of acoustic dilution/concentration when applicable, the third column specifies the dilution required for spectrophotometer measurement (absorption below 1), and the last column outlines the final dilution factor. This information is presented using yeast suspension as an example

Yeast samples	Estimation of acoustic dilution	Dilution for absorbance measurement	Final dilution factor
Calibration	0	Range from 10 to 100	Range from 10 to 100
Control (no acoustics)	0	20 ×	20 ×
Enriched suspension (center)	Between 0.5 × and 0.75 ×	100 ×	Between 50 × and 75 ×
Diluted suspension (side)	Between 2 × and 3 ×	20 ×	Between 40 × and 60 ×

**Table 3:**
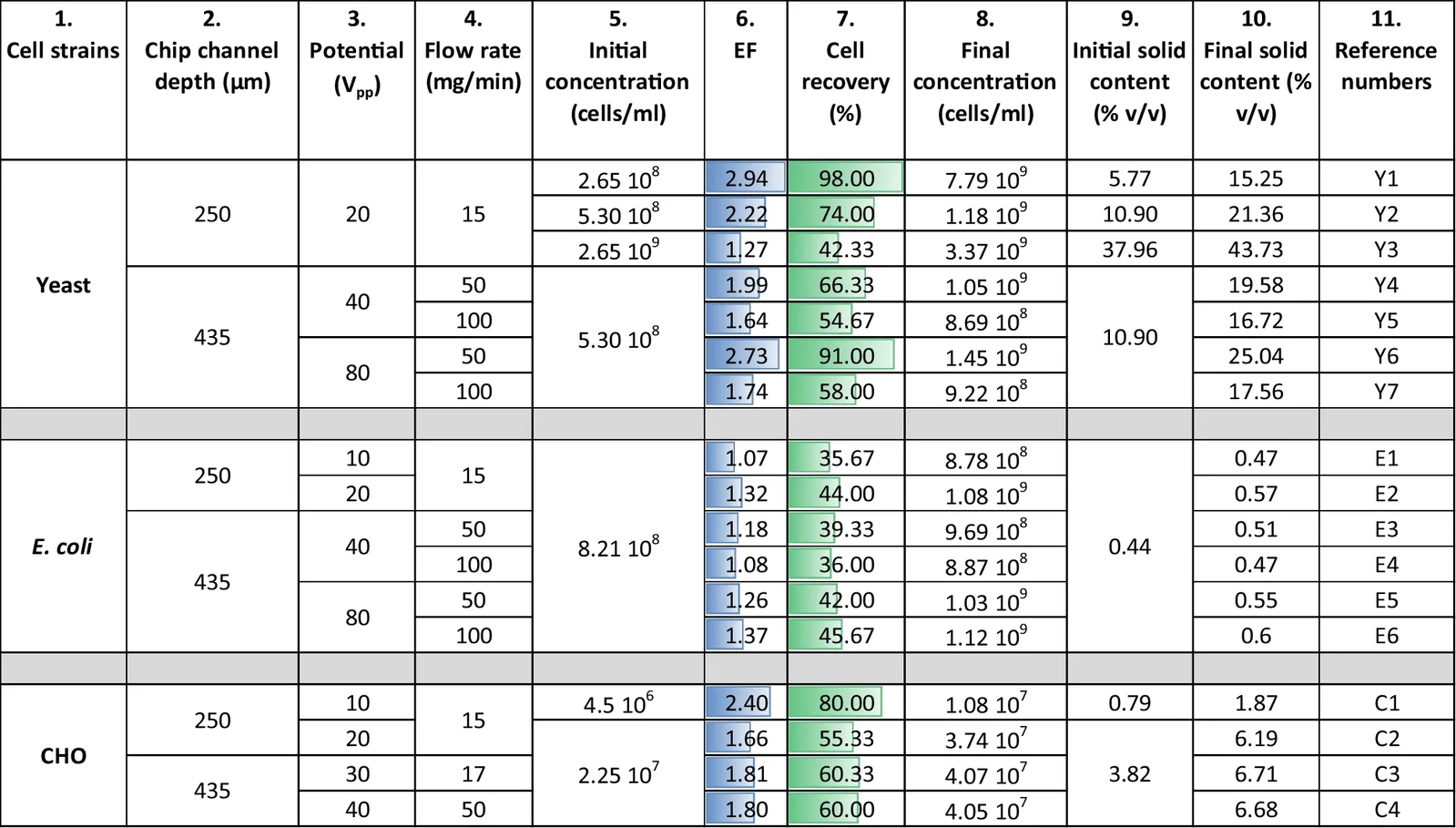
$\mathrm{EF}$ values, fractions of cells recovered and finals suspension concentration (column 6 to 8) resulting from the different experiments with the corresponding parameters (column 1 to 5), initial and final calculated solid content (column 9 and 10), and reference numbers (column 11)

All samples underwent proper dilution to align with the calibration curve. Table 2 provides details on the various dilutions needed to establish the calibration curve, outlines the sample preparation for spectrophotometer measurement, and includes the calculations to determine the real concentration of the different samples.

### Acoustic enrichment

**Fig. 5:**
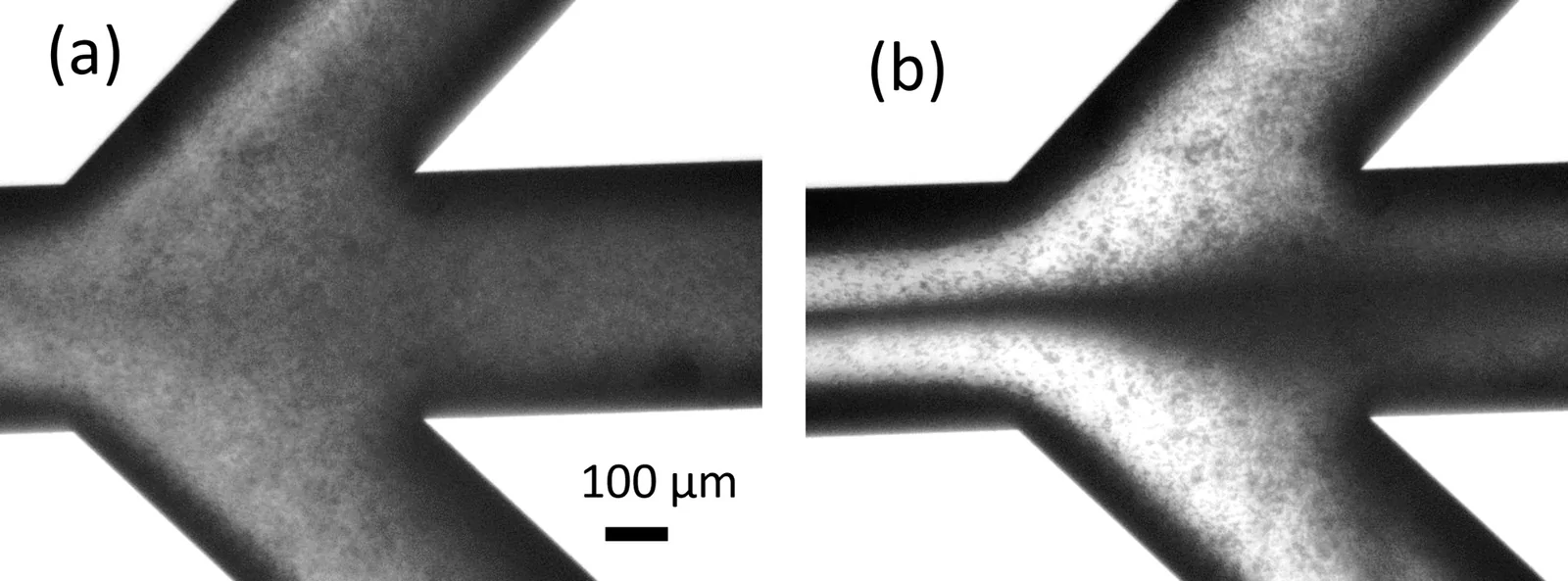
Focusing behavior yeast suspension stream in the main channel split **a** without and **b** with acoustic focusing. With acoustics, cells focused in the center of the main channel, were preferentially flowing through the central channel. The liquid, flowing though the two sides channel, had a lower cell content. Yeast suspension with a concentration of 2.65 10^8^ CFU/ml, in a 250-µm deep channel, with a density of 15 mg/min, with application of respectively 0 V_pp_ (**a**) and 20 V_pp_ (**b**) (sample Y1 in Table 3)

As an example, an image of the chip region depicted in Fig. 2 (iii) containing yeast cells is given in Fig. 5a without acoustic and Fig. 5b with acoustic focusing. Without acoustics, the cells equally leave the chip through the three outlets. With acoustic standing wave, cells, previously focused in the center of the main channel, preferentially left the chips through the central outlet. The liquid leaving through the side channels had a low cell content.

Assuming that the liquid flow was identical at the three outlets (experiments were only conducted where the difference was below 1%), the enrichment factor $\mathrm{EF}$, has been defined to characterize the process efficiency as the ratio of final to initial concentration. The $\mathrm{EF}$ was subsequently calculated with:

5 $$\begin{array}{cc}\mathrm{EF}=\frac{{C}_{c}}{{C}_{i}}& \text{with }\left(\mathrm{EF}\le 3\right)\end{array}$$

$$\mathrm{EF}>1 :\mathrm{enrichement}$$

$$\mathrm{EF}=1 :\text{ no change}$$

$$\mathrm{EF}<1 :\mathrm{dilution}$$

with ${C}_{i}$ the concentration of the initial suspension and ${C}_{c}$ the concentrations of the suspension at the central outlet.

The fraction of cells recovered, ${R}_{\%}$ (collected to the central outlet) was calculated using:

6 $${R}_{\%}=\frac{100\;\mathrm{EF}}{3}$$

Factor 3 comes from the division of the main channel in three outlets of same dimension (Fig. 2b). The enrichment resulting from the acoustic process, is influenced by the different parameters that are detailed in Table 3. The five first columns detail the experimental conditions, columns 6 to 8 give the results, columns 9 and 10 indicate the initial and final calculated solid content, and in the last column reference numbers are given.

As an example of a calculation, in the second line, an enrichment factor of 2.22 results in a final concentration of 5.30 10^8^ × 2.22 = 1.18 10^9^ cells/ml, which corresponds to a solid content of 21.36%.

The first part of Table 3 concerns the yeast suspension. The impact of concentration, flow, and potential on the concentration factor was analyzed. At lines Y1, Y2, and Y3, the solid content increased from 5.77 to 37.96%, it resulted in a decrease of the $\mathrm{EF}$ value from 2.94 to 1.27. Comparing lines Y4–Y5 and Y6–Y7, an improvement of the process was observed for a decreasing of the flow rate, from 50 and 100 mg/min. This was noted at two potentials, 40 *V*_pp_ (Volt peak to peak) and 80 *V*_pp_. Finally, this increase of potential resulted in an increase of the $\mathrm{EF}$ value, as indicated in lines Y4–Y6 and Y5–Y7.

The acoustic process had a low efficiency on the *E. coli* suspension. Indeed, the highest $\mathrm{EF}$, all parameters included, was 1.37. Nevertheless, the positive effect of increasing potential on $\mathrm{EF}$ value was observed comparing lines E1–E2, E3–E5, and E4–E6. They were measured with increasing potential, from 10 to 20 *V*_pp_ and 40 to 80 *V*_pp_ at 50 and 100 mg/min, respectively. The variation of the $\mathrm{EF}$ value with flow was relatively low with *E. coli*. When linking E3–E4 and E5–E6, the opposite effect was observed. An increase of 0.10 appeared in the first pair and a decrease of 0.11 in the second.

For the CHO suspension, despite an increase of the potential from 10 to 20 *V*_pp_, a decrease of the $\mathrm{EF}$ value of 0.74 was noted when concentration was multiplied by five, between lines C1 and C2. Finally, C3 and C4 displayed respective $\mathrm{EF}$ values of 1.80 and 1.81 emphasizing the increasing potential counterbalancing the effect of increasing flow.

## Discussion

The different parameters are further discussed in this section. The three cell strains represented a variation of the acoustic contrast factor and cell dimensions. Both chips had a different channel depth. Three other parameters were screened: electrical potential applied on the PZT, suspension concentration, and liquid flow rate.

### Cell strains

Using an identical chip and applying the same voltage and flow, the process on *E. coli* suspension systematically resulted in a lower $\mathrm{EF}$ value compared to yeast and CHO suspensions. This is clear when comparing couples E2–Y2, E3–Y4, E3–C4, E4–Y5, E5–Y6, and E6–Y7. The only differences between these pairs resided in cell strains and their respective solid content. The yeast and CHO suspensions had a higher solid content, 10.90 and 3.82% v/v respectively, compared to *E. coli* with 0.44% v/v. E2 and Y3 could be further compared. They resulted in close $\mathrm{EF}$ values of 1.32 and 1.27 with respective solid content of 0.44 and 37.96%. To reach a higher $\mathrm{EF}$ value, *E. coli* must therefore display a solid content 188 times smaller. From literature, an increasing solid content results in a lower $\mathrm{EF}$ value (Karthick and Sen 2016, 2017; Ley and Bruus 2016). The opposite effect was observed here. The diameter of *E. coli* (Willey et al. 2017) is situated in the range of 0.5 to 1.5 µm and a critical particle diameter of about 1.4 µm was calculated for polystyrene particles in water (Barnkob et al. 2012). The lower process efficiency with *E. coli* can therefore be attributed to the small dimension of the cells and/or to their low contrast factor. The elongated shape could also influence the result as well, this is further discussed below with the influence of the shear rate.

Yeast and CHO suspensions can also be compared, in lines Y1–C2, measured in the same chip with the same voltage and flow. The $\mathrm{EF}$ value of C2 was lower, which is against the expectation based on cell dimensions and solid content. Indeed, CHO cells have a higher dimension, 15 µm versus 5 µm for yeast, and a lower concentration. This result indicates that the cell contrast factor of yeast is higher than the one of CHO.

Next to its dimensions, the radiation force on cells is characterized by the acoustic contrast factor which depends on three parameters (Barnkob et al. 2012; Muller et al. 2012), $\widetilde{\kappa }, \widetilde{\rho ,} \mathrm{and} \widetilde{\delta }$. They result from the interaction between the surrounding medium and cell properties. It includes the impact of the local concentration along the channel width when particles are focused.

Building upon literature, different contrast factors are expected and indeed observed between the three cell strains. This disparity in contrast factors is supported by the marginal variations in size and properties among cells of the same strain at different developmental stages, compared to the distinctions among cells of different strains (Sharpe 1988). For instance, Olofsson et al. (2020) explored the separation of dead and living cells based on their contrast factor differences by increasing the medium density. Dead and living cells were focused near the nodes and antinodes, respectively. This concept was subsequently applied to gauge the contrast factors of three cell strains. Augustsson et al. (2010) determined the acoustic contrast factor for undifferentiated and 4-day-differentiated human embryonic ventral mesencephalic cells, revealing contrast factor values of 0.04 and 0.07, respectively.

$\widetilde{\kappa }$ and $\widetilde{\rho }$ are respectively the ratio of compressibility and the density between the liquid and the cell. The relative densities of yeast (Baldwin and Kubitschek 1984), *E. coli* (Woldringh et al. 1981), and CHO (Anderson et al. 1970) respectively fall within the ranges of $\widetilde{\rho }=1.1094 to 1.1173$, $\widetilde{\rho }=1.080 \mathrm{to} 1.100$ and $\widetilde{\rho }=1.0557 \mathrm{to} 1.0606$. The cell density however varies with conditions as the stage of development and the surrounding medium composition (Anderson et al. 1970; Woldringh et al. 1981; Baldwin and Kubitschek 1984). Cell compressibility is also influenced by several parameters as pH or surrounding molecules (Weinberger and Kulozik 2022). In the literature, an acoustofluidic device was developed to measure the compressibility of cells, considered as a marker for cancer diagnosis, by analyzing the cell motion trajectory under acoustic force (Fu et al. 2021, 2022).

Concerning the density and compressibility of the surrounding liquid, the standard CHO suspension, provided by the pharmaceutical partner, was introduced in the chips as received. In contrast, yeast and *E. coli*, delivered in higher concentrations, were diluted. The growing medium initially contained a proportion of nutrients, increasing the liquid density. Nutrients are, however, consumed by cells in unknown quantities influencing solution viscosity and density (Poon 2022). As a result, it impacts the contrast factor and therefore the acoustic enrichment process.

Finally, as the solid content increases, cell interactions become significant, impacting liquid properties. Ladd (1990) measured an increasing density of the medium containing an increasing content of hard spheres. Experimentally, as cells were focused towards the center of the channel, a concentration gradient grew, resulting in an increasing local medium density. Consequently, the density ratio ($\widetilde{\rho }$) between the cells and the medium gradually decreases towards the channel center.

$\widetilde{\delta }$ is the ratio between the acoustic boundary layer thickness and the cell radius, both influenced by the cell strains and their concentration. The boundary layer thickness increases with the liquid viscosity (Barnkob et al. 2012; Muller et al. 2012). Together with density, Ladd (1990) measured an increasing viscosity with higher solid content. As discussed in the “Materials and method” section, cell dimensions are influenced by different parameters such as constriction or division cycle (Trueba and Woldringh 1980). They furthermore exhibit different shapes, such as cylinder for *E. coli*.

It is interesting to note that some cells present adherent properties. In this case, two scenarios are possible. Firstly, if cells exhibit a propensity to adhere to the surface of the chip, it may have a detrimental impact on the process. Conversely, if cells demonstrate an affinity for adhering to each other, the process could potentially become more efficient. In this scenario, the cells act as larger particles, enhancing the radiation force and diminishing the observed repelling force between them.

### Channel depth

**Fig. 6:**
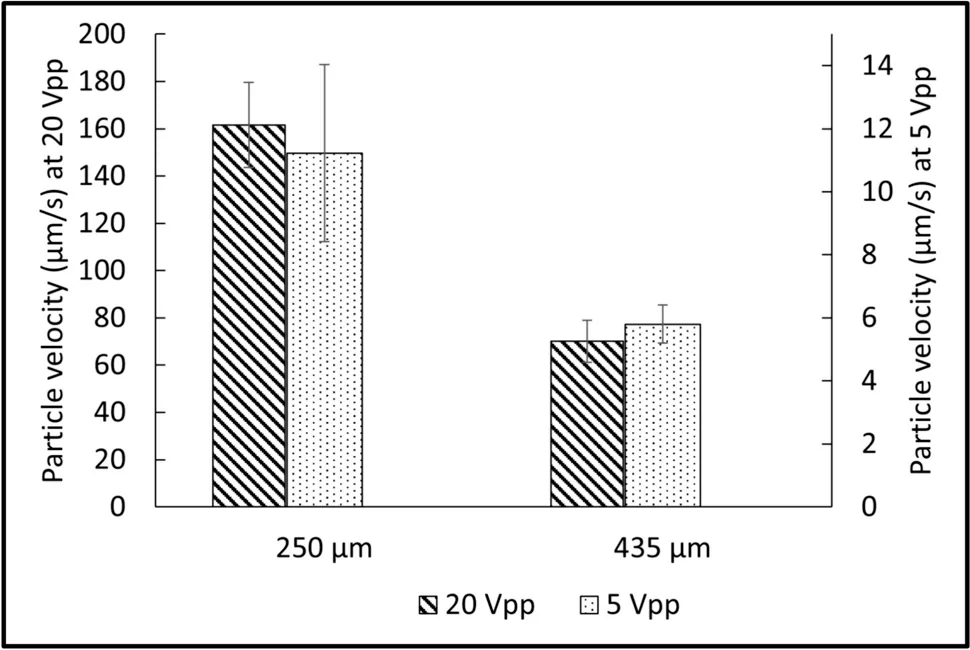
Average radiation velocity of the particles between *x*=$\frac{1}{8}w$ and $\frac{3}{8}w$ (with *x* the position along the channel width and *w* the channel width) as a function of the channel depth

The radiation velocity of PS particles, focused at potentials of 5 and 20 *V*_pp_, is represented in Fig. 6 for 250- and 435-µm deep channels. At low concentration, the absence of interactions between particles was assumed. With 20 *V*_pp_ (left scale) as well as with 5 *V*_pp_ (right scale), the velocity was higher for the chip with a 250-µm deep channel. While the channel depth increased by a factor of 1.74 (250 to 435 µm), the velocity increased by a factor of 2.12.

Interestingly, this phenomenon contradicted the numerical simulations conducted by Spigarelli et al. (2020), who observed an augmentation in radiation velocity as channel depths increased, particularly when the aspect ratio transitioned from below one to higher than one. The numerical simulations generally introduce the BAW as a vibrating wall with an amplitude, a frequency, and a distance which is identical in the different studied chips. In practice, however, the vibration is induced by the PZT device and these parameters changed with the channel depth, as more volume needs to be actuated with the same power. The total acoustic energy introduced in the system was identical (in electrical potential) for both tests, i.e., the acoustic energy per volume/surface unit was higher in the chip with a 250-µm deep channel. This effect was proportional when 5 and 20 *V*_pp_ were applied.

### Electrical potential

The effect of the potential on the radiation is first discussed in this section. Next, its secondary impact on temperature and cavitation is described.

As detailed in Table 3 and in the beginning of this discussion, for the three strains, an increase in potential resulted in improving acoustic focusing and concomitantly the $\mathrm{EF}$ value. The influence of the potential on radiation is discussed in light of the graph in Fig. 6. The potential applied on the PZT was increased from 5 to 20 *V*_pp_. In response, the radiation velocity increased by a factor of 14.4 and 12.1 in channels depths of 250 and 435 µm, respectively. In theory, the radiation velocity is directly proportional to the acoustic energy density (Barnkob et al. 2012). The acoustic energy density, in turn, is proportional to the square of the acoustic pressure which is proportional to the voltage applied on the PZT (Barnkob et al. 2010). An increase of the voltage, by a factor of 4, should therefore increase the radiation velocity, by a factor of 16. The discrepancy between the experimental and the expected velocity can be explained by a loss of energy in the glycerol coupling layer as well as by dissipation through heating of the system.

Experimentally, without the cooling system, voltages above 20 *V*_pp_ generated cavitation bubbles within few seconds. Additionally, an increase of the temperature was observed. This secondary effect of increasing potential was undesirable, causing disturbance in the flow and potentially giving rise to cell damage (Oyama et al. 2021). Acoustic cavitation is a vaporization process that arises from the pressure reduction below some critical value (Leighton 1994; Brennen 2013). In pure water, the cavitation will be triggered under a very strong tension, at 27 MPa at 10 °C (Herbert et al. 2006). In our system, the acoustic pressure amplitude P is in the 0.1 MPa range (Ley and Bruus 2016). This value can also be calculated with Eq. 7 (Oyama et al. 2021):

7 $$P=2\pi f{\rho }_{0}cA$$

with $f,{\rho }_{0},c$, and A the frequency, the liquid density, the speed of sound, and the PZT static displacement, respectively. At 40 *V*_pp_, the static displacement is 0.0116 µm (APC International Ltd). In water, with 2 MHz, it results in a pressure amplitude of 0.218 MPa.

In both cases, the pressure amplitude is far below the vapor pressure required for cavitation. However, it is hypothesized that cavitation can occur in suspensions saturated with ambient air, even with low intensity acoustic waves. This is corroborated by previous studies where cavitation occurred with a frequency of 1 MHz at similar voltages and with a pressure amplitude of 0.169 and 0.285 MPa (Nguyen et al. 2017; Oyama et al. 2021). In addition, Herbert et al. (2006) reported some discrepancies between experiments and theoretical models. Cell media may also contain surfactants, which reduce the surface tension and facilitate gas bubble formation (Rosen 2004).

Finally and most importantly, cavitation intensifies with temperature (Lide 2005; Magaletti et al. 2021). In pure water, cavitation was triggered at 2 MPa at 50 °C (Herbert et al. 2006) (compared to 27 MPa at 10 °C as mention above). Next to the curves given in Fig. 3, the temperature of the thermocouple, placed on the PZT was recorded. It reached 29.8, 42.5, and 68.3 °C with voltages of 20, 40, and 80 *V*_pp_, respectively, after 120 s. This explained the appearance of cavitation bubbles in the experiments and dictated the necessity of temperature control. The latter assumption was also supported by the suppression of cavitation when the cooling system was used.

### Influence of diffusion

In Table 3, it is evident that the $\mathrm{EF}$ value decreases with an increase in solid content for yeast and CHO suspensions, as illustrated by samples Y1, 2, and 3. Similarly, the $\mathrm{EF}$ value decreases with an increase in flow rate, as observed in samples Y6 and 7.

Upon introducing cell suspensions into the chip depicted in Fig. 2b, two opposing forces act to transport them laterally: the radiation force and diffusion. Experimental observations revealed an instantaneous spreading of the focused bandwidth when the acoustic field was switched off. This suggests that in suspensions with high solid content in flow, the diffusion coefficient calculated using the Stokes–Einstein law is insufficient to explain cell spreading along the channel width.

Karthick and Sen (2016) developed a system analogous to the one described in this paper for enriching red blood cells. They measured the relative diffusion times of red blood cell suspensions under flow conditions, confirming an increase in diffusion velocity with flow rate. This phenomenon, attributed to shear-induced diffusion (SID) (Li et al. 2007; Lopez and Graham 2007; Rusconi and Stone 2008; Karthick and Sen 2016, 2017), is a natural mechanism of migration raising from a collective behavior of particles (Drijer and Schroën 2018).

The theoretical model, detailed by Karthick and Sen (2016 and 2017), used red blood cells to observe acoustic focusing. The paper describes suspension behavior when particle–particle interactions can no longer be ignored. In this model, diffusion is initially increased by the rise in solid content. As the solid content increases, viscosity and, concomitantly, mobility decreases (Krieger and Dougherty 1959; Ladd 1990). This viscosity increase was also demonstrated for yeast and *E. coli* suspensions (Reuß et al. 1979; Toda et al. 1998; Gachelin et al. 2013). Second, as the flow rate increases, a new term, shear-induced diffusion (SID), emerges. SID is characterized by a diffusion coefficient increased by several orders of magnitude compared to the standard diffusion coefficient (Lopez and Graham 2007). This term also increases in the case of soft particles, such as red blood cells, yeast, *E. coli*, or CHO cells.

Equation 8 describe the particle flux due to diffusion (Leighton and Acrivos 1986, 1987; Phillips et al. 1992; Tan et al. 2012)

8 $${J}_{\mathrm{SID}}={D}_{\varphi }\nabla \varphi +{D}_{\gamma }\nabla \gamma$$

${D}_{\varphi }$ and ${D}_{\gamma }$ are respectively the diffusion coefficient due to the gradient in volume fraction φ and shear rate γ. These diffusion coefficient are function of particle dimension, shear rate, concentration, shape, and deformability (Karthick and Sen 2016, 2017). The diffusion coefficient is extensively discussed in a theoretical model developed by Ley and Bruus (2016) and further discussed in the Supplemental information. Influence of the concentration on viscosity, diffusion and mobility as a function of the volume fraction are plotted in Supplemental Fig. S1 with data generated by Ladd (1990). The radiation time and the diffusion time were calculated applying conditions listed in Supplemental Table S1. The dimensionless ratio of these two characteristic times defined as *Τ* value, expressed in Eq. S11, gives an indication of which phenomenon dominates. *T* value was plotted as a function of the solid content in Supplemental Fig. S2.

Rusconi and Stone (2008) measured the SID coefficient as a function of the shear rate for plate-like clay particles with a 1-µm radius at different volume fraction. The SID coefficient was higher for plate-like compared to spherical particles, showing the importance of anisotropy. Clay particles have different properties compared to cells, but this study highlights the importance of the shape. Next to its suspected low contrast factor, the elongated shape of *E. coli* could therefore cause an increased SID explaining the lower efficiency of the acoustic process.

In the context of suspensions containing deformable particles like drops, vesicles, or cells, the distortion of objects during shear introduces asymmetry. When the trajectories of two particles intersect under shear flow, there is a repulsive effect, leading to an increased cross-streamwise distance between the objects after their encounter (Podgorski et al. 2011).

This phenomenon probably influences the focusing of cells and to a higher extent for *E. coli* presenting an elongated shape.

As a result, the SID resulted in a maximum solid content for an enrichment higher than two with the current device geometry. For the applied experimental conditions, this limitation was defined for the yeast suspension between 5.30 × 10^8^ and 2.65 × 10^9^ cells/ml. The limit for the CHO suspension is between 2.25 10^7^ and 4.5 10^6^ cells/ml. The limit was not reached for the case of the tested *E. coli* suspension. The influence of the flow and the shear rate is further discussed in the second section of Supplemental information.

### Influence of secondary forces

As the concentration of cells or particles increases while migrating through the center, there is a corresponding increase in the secondary radiation force due to cell–cell interactions (Petersson et al. 2004; Collino et al. 2015). This secondary force becomes attractive when particles tend to form clusters. The formation of these clusters results from the combined effects of both primary and secondary acoustic radiation forces. However, this phenomenon is observed specifically for hard and isotropic particles (Groschl 1998; Kuznetsova and Coakley 2007; Dong et al. 2019). In the case of soft particles like cells (Weiser et al. 1984; Petersson et al. 2004), the secondary force becomes repulsive, and this effect intensifies when rod-like particles, such as *E. coli*, are used (Collino et al. 2015).

As a conclusion of the whole study, an acoustofluidic device designed to concentrate living cell suspensions in continuous mode has been evaluated. The process is characterized by the introduced enrichment factor, and various parameters have been examined. Three distinct types of cell suspensions were infused into chips with two different microchannel depths. The variation of voltage, suspension concentration, and flow rates were tested and discussed.

The application of the acoustofluidic method yielded promising results that warrant further optimization for economically viable applications. The study highlighted complex interactions among the parameters.

Different cell strains exhibited varied responses to acoustic radiation due to differences in dimension, shape, density, and compressibility. The influence of cells on their surrounding medium, coupled with each suspension’s unique acoustic contrast factor, contributed to the observed differences. Despite a lower concentration and larger dimensions, CHO cell suspensions were less efficiently focused compared to yeast cells. The *E. coli* suspension exhibited a low response to the radiation force, potentially attributed to its small dimensions and an elongated shape that increased shear-induced diffusion.

The channel depth influenced the acoustic energy density introduced into the suspension. At similar voltages, chips with deeper channels had a lower amount of energy introduced per unit volume, and this effect was proportional at two different voltages.

The voltage applied to the PZT directly influenced cell focusing by increasing the acoustic energy density (radiation force). Theoretically, a fourfold increase in voltage should result in a 16-fold increase in particle radiation velocity. However, experimental results showed a slight discrepancy explained by energy loss, such as through system heat dissipation.

As the suspension concentration increased and cells interacted, viscosity increased, and cell mobility decreased. Migration of particles toward the center of the channel created a concentration gradient, reducing process efficiency. This phenomenon was however insufficient to justify the experimental results, suggesting a strong influence of the flow rate on the concentration process.

The flow rate negatively impacted focusing efficiency through a rising of shear-induced diffusion. It increases mass transfer by diffusion against the concentration gradient. This phenomenon depends on shear, solid content, shape, and cells deformability.

Finally, repulsive secondary radiation forces are rising with increasing concentration. Again, the *E. coli* elongated shape worsens the enrichment efficiency.

In conclusion, the device demonstrated promising performance for industrial applications. Shear-induced diffusion, however, limited concentration and flow rate in the current design. Improving chip geometry could extend these limits while maintaining optimal process performance.

## Supplementary Information

Below is the link to the electronic supplementary material.

Supplementary file1 (PDF 809 KB)

## Data Availability

The data that support the findings of this study are available from the corresponding authors upon reasonable request.
