# Supplementary material for: Exploring operational boundaries for acoustic concentration of cell suspensions
Source: Appl Microbiol Biotechnol. 2024 Jun 19;108:387. doi: 10.1007/s00253-024-13215-1 (PMC11186915; doi:10.1007/s00253-024-13215-1)
Supplement: Supplementary file 1 [file 253_2024_13215_MOESM1_ESM.pdf]

Electronic supplementary information (ESI)

Journal name: Applied Microbiology and Biotechnology

Manuscript title: Exploring operational boundaries for acoustic concentration of cell suspensions

Amaury de Hemptinne[1*], Pierre Gelin[1], Ilyesse Bihi[1], Romain Kinet[2], Benoit Thienpont[2], Wim De Malsche[1*]

[1]µFlow Group, Department of Chemical Engineering, Vrije Universiteit Brussel, 1050 Brussels, Belgium

[2]GSK, Rixensart, Belgium

*Corresponding authors: E-mail addresses: amaury.de.hemptinne@vub.be (A. de Hemptinne), wim.de.malsche@vub.be (W. De Malsche).

Influence of suspension concentration

A theoretical model, detailed by Ley and Bruus (2016), describes the suspension behavior when particle-particle interactions can no longer be ignored. Briefly, as the solid content increases, the viscosity increases and the diffusion coefficient (and concomitantly the mobility) decreases (Krieger and Dougherty 1959; Ladd 1990). When a suspension of particles is introduced into the microfluidic chip, two opposite effects transport particles in the lateral direction. With radiation force, standing acoustic waves focus particles towards the center of the main channel, forming a gradient of concentration. As a result, the high concentration, in the center of the channel, is characterized by high viscosity and low mobility resulting in a decreasing radiation velocity. Simultaneously, according to the Fick's second law of diffusion (Dhont 1996; Cussler 2007), a higher concentration gradient results in an increase in diffusion.

These two effects can be described in term of lateral particles flux J_l, with Eqs. S1 and S2 (Ley and Bruus 2016).

$$J_l = J_{diffusion} + J_{radiation} \qquad Eq. S1$$

$$J_l = -D_\varphi(C_p)\nabla C_p + C_p \upsilon(C_p) F_r \qquad Eq. S2$$

with F_r the acoustic radiation force, $D_\varphi(C_p)$ and $\upsilon(C_p)$ the diffusion coefficient and the mobility of particles respectively, both depending on particles concentration C_p.
The hydrodynamic interactions between particles are included in the model through the particle concentration C_p , and the volume fraction V_f, linked together in Eq. S3.

$$V_f = \frac{4}{3}\pi r_p^3 C_p \qquad Eq. S3$$

with r_p the particles radius.

The diffusion coefficient and the mobility were expressed in Eqs. S4 and S5 (Ley and Bruus 2016).

$$D_{\varphi}(C_p) = \psi_D(V_f)D_0 \qquad Eq.S4$$
$$\upsilon(C_p) = \psi_\upsilon(V_f)\upsilon_0 \qquad Eq.S5$$

with D_0 and υ_0 the diffusion coefficient and the mobility of particles in diluted suspension (no particles-particles interactions). ψ_D and ψ_υ are the diffusion and the mobility concentration correction factors respectively. Compiling Eqs. S1 to S5 results in Eq. S6.

$$J_l = -\psi_D(V_f)D_0\nabla C_c + C_c\psi_\upsilon(V_f)\upsilon_0 F_r \qquad Eq.S6$$

The diffusion coefficient and the mobility correction factors were experimentally measured by Ladd (1990) for hard spherical particles (Fig. S1). In both cases, a decrease following a fourth-order equations (Ley and Bruus 2016) with the volume fraction was observed.

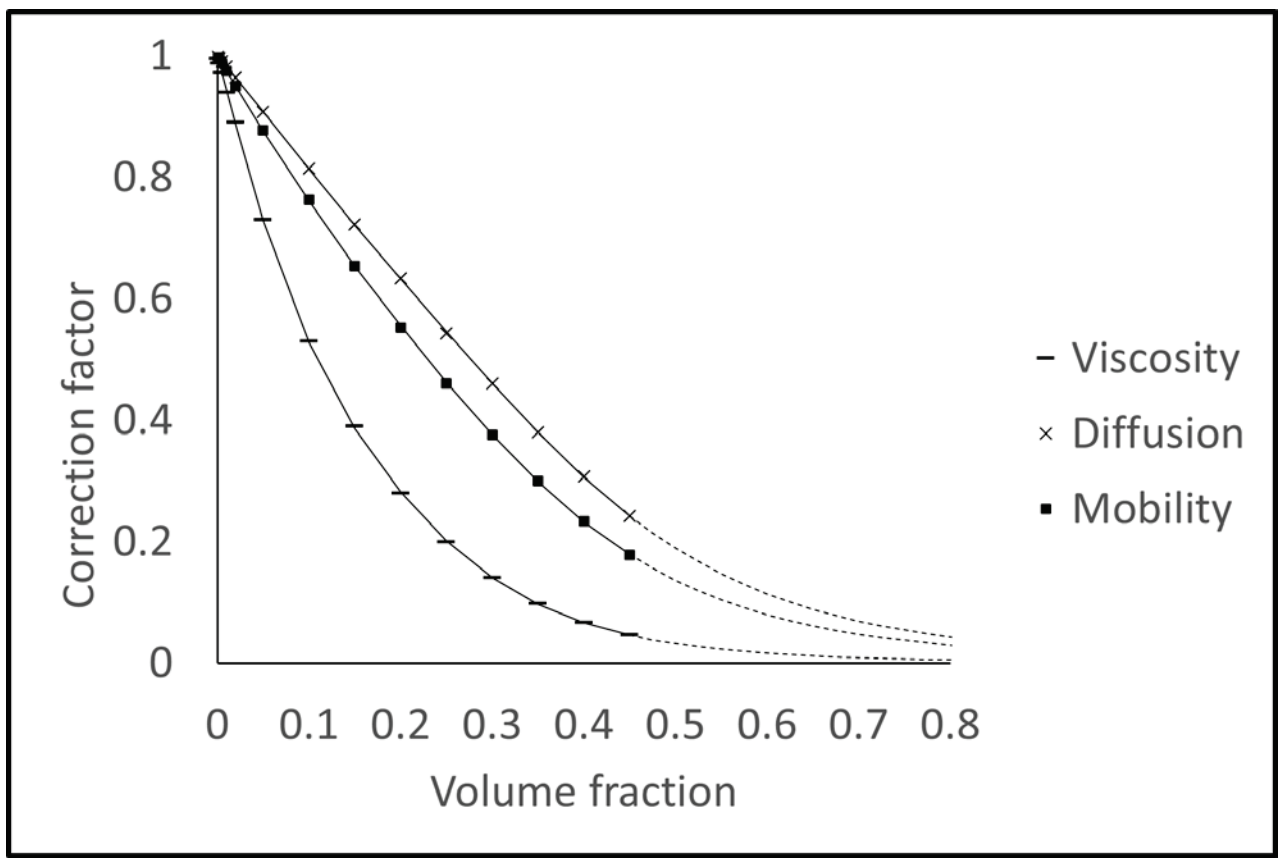

Fig. S1: Correction factors for particles diffusion coefficient (×), mobility (▪) and viscosity (-) as a function of the volume fraction. The lines correspond to fitting equations based on the 14 data points from Ladd (1990).

Next to diffusion and mobility, Ladd (1990) described the influence of the volume fraction on the viscosity (Eq. S7 (Ley and Bruus 2016)). A high solid content results in a high viscosity:

$$\mu(V_f) = \psi_\mu(V_f)^{-1}\mu_0 \qquad Eq.S7$$

with ψ_μ the viscosity concentration correction factor, plotted in Fig. S1, and μ_0 the viscosity of the diluted suspension.

In order to compare, diffusion and radiation, they can be expressed them in terms of the characteristic time τ. A low time scale corresponds to a fast, thus dominating process. The characteristic diffusion time τ_d was introduced in the literature (Chruickshank-Miller 1924; Dhont 1996). When adapted to the current chip, this yields Eq. S8.

$$\tau_d = w^2/2D_\varphi \qquad Eq.S8$$

with w the channel width. The characteristic radiation time is calculated with Eq. S10 which results from Eq. S9 and second Newton's law equalizing the radiation and the drag forces.

$$\tau = \frac{distance}{velocity} = \frac{w/2}{v(x)} \qquad Eq.S9$$

$$\tau_r = 3\pi\mu r_p w / F_r\,(x) \qquad Eq.S10$$

with v the radiation velocity, μ the dynamic viscosity and r_p the particles radius. The dimensionless ratio of these two characteristic times T, expressed in Eq. S11, gives an indication of which phenomenon dominates. If T is high, radiation dominates and the acoustic process is efficient. If T is low, diffusion dominates.

$$T = \tau_d / \tau_r = w\, F_r(x) / 6\pi\mu D_\varphi r_p \qquad Eq.\ S11$$

The diffusion and radiation characteristic times for hard particles were calculated, taking into account the viscosity and the diffusion correction factors (Ladd 1990; Ley and Bruus 2016). Experimental conditions of this work were applied to calculate the *T* value as a function of the solid content. The acoustic parameters and the radiation force were chosen and calculated with the work of Barnkob et al. (2012) and Muller and Bruus (2014). Table S1 presents an example of calculation of one *T* value. Fig. S2 presents the evolution of the *T* value as a function of the solid content.

This resulted in a *T* value in the range of 10^5 to 10^7, indicating radiation dominance. As the solid content increases, the diffusion time increases due to the concentration gradient, but the radiation time also increases through the viscosity correction factor. Even with a solid content of 50%, the resulting *T* is 3.96 10^6.

These values indicate that acoustic focusing is efficient. Experimental results at low concentration confirm this calculation but it becomes inaccurate at high solid content. This calculation is valid for hard sphere suspension; nevertheless, the order of magnitude of *T* indicates that it is influenced by another phenomenon, potentially linked to the shear rate and discussed in the next section.

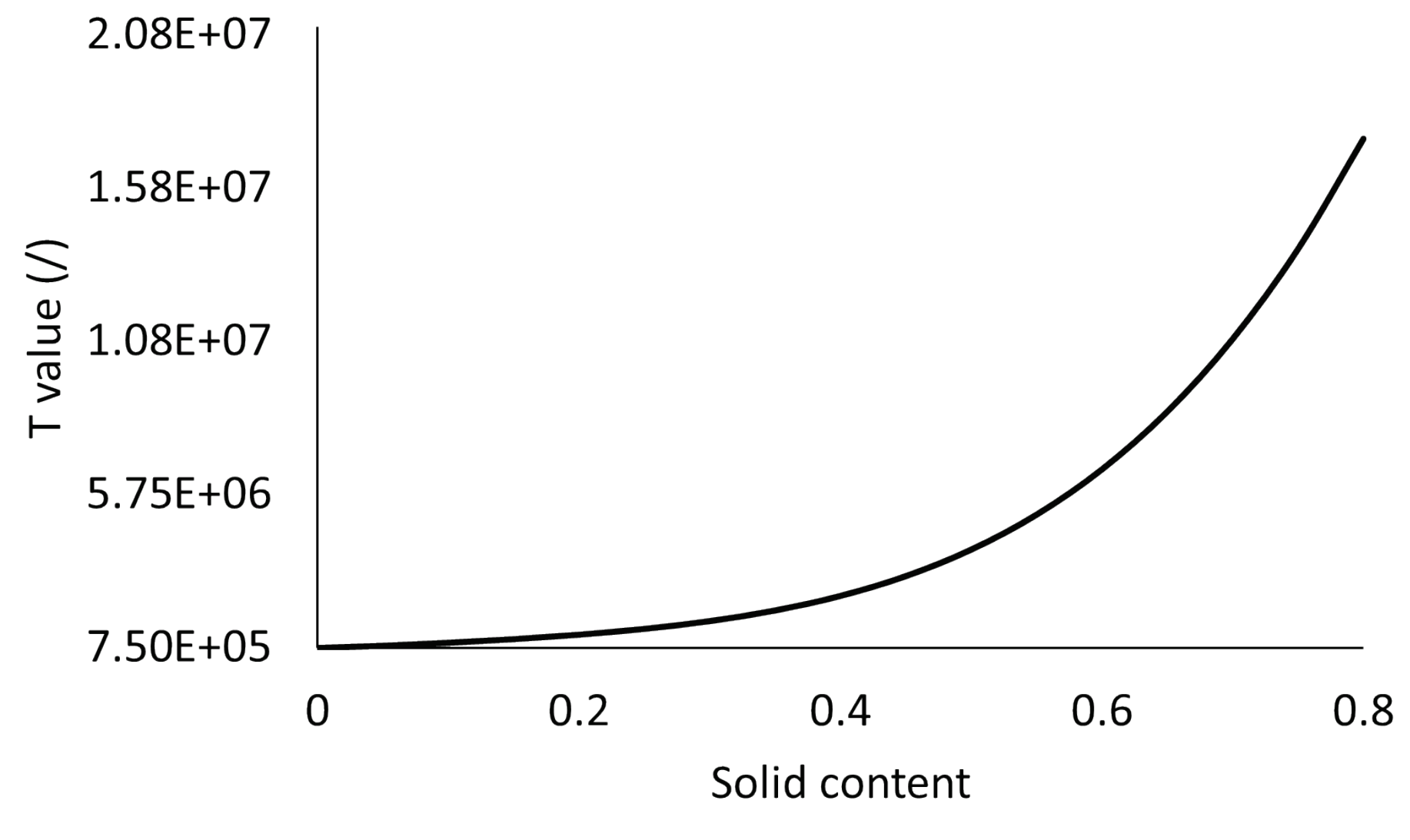

Fig. S2: *T* value as a function of the solid content for 5 μm PS particles in water for a single node system in a 375 μm wide channel considering the viscosity and diffusion correction factor. An acoustic contrast factor of 0.165 and an acoustic energy density of 20 J/m³ have been applied (Ladd 1990; Barnkob et al. 2012; Ley and Bruus 2016).

Table S1: Input parameters used to calculate the T value.

**Liquid viscosity**	1	μ, mPa.s
**Volume fraction**	0.1	V_f
**Viscosity correction factor**	0.76	ψ_μ
**Diffusion correction factor**	0.85	ψ_D
**Number of node**	1	n
**Channel width**	375	d, μm
**Wavelenght**	0.00075	λ, m
**Frequency**	2	f, MHz
**Acoustic contrast factor**	0.165	θ
**Particle diameter**	5	μm
**Acoustic energy**	20	E_{ac}, J/m^3
**Velocity of sound in liquid**	1497	c, m/s
**Diffusion coefficient**	$7.48\ 10^{-14}$	D
**Diffusion time**	$9.40\ 10^{5}$ s	τ_D, s
**Radiation time**	1.07 s	τ_r, s
**T**	$8.79\ 10^{5}$	

Influence of the flow and the shear rate

As indicated in the results, an increase in flow rate resulted in a decrease in the process efficiency for the three strains.

The flow rate has two main effects on the process. First, when it is increased, the residence time of cells in the chip decreases i.e., they have less time to move towards the center of the channel.
The advection is characterized by the transport of particles by the surrounding liquid flow in the longitudinal direction. A characteristic time τ_a associated with this flux is expressed in Eq. S12.

$$\tau_a = L/u \qquad Eq.S12$$

L is the width of the PZT, corresponding to the longitudinal distance over which the radiation force is active on particles. u is the cells' velocity in the longitudinal direction, considered as equal to the surrounding liquid velocity. A characteristic time of 1.47 s was calculated with a PZT of 1.5 cm and liquid flow of 100 mg/min in a microchannel of 375x435 μm (assuming 1 mg=1 μL). This advection time is of the same order of magnitude as the radiation time calculated in Table S1.

By adjusting parameters, as voltage and flow, the dominance of migration over advection can be established, resulting in an efficient acoustic process. The ratio of advection time to radiation time is represented by the dimensionless Strouhal number St:

$$St = \tau_a/\tau_r \qquad Eq.S13$$

The flow can also influence the focusing of particles through a second phenomenon. In laminar flow, as the shear rate increases, the diffusion flux in perpendicular direction to the flow also increases, counteracting the radiation force.

Experimentally, as well as in literature (Karthick and Sen 2016), when switching off the acoustic field, an instantaneous spreading of the focused bandwidth was observed. This indicates that, for a flowing suspension, the diffusion coefficient calculated with the Stokes-Einstein law is no longer valid. This observation was supported by measurements of the relative diffusion times for flowing and non-flowing red blood cell suspensions (Karthick and Sen 2016). When particles are set in motion by the surrounding liquid, a phenomenon named shear induced diffusion (SID) arises (Lopez and Graham 2007; Karthick and Sen 2016; Karthick and Sen 2017). This diffusion is characterized by a diffusion coefficient that increases by several orders of magnitudes compared to the standard coefficient of particles (Lopez and Graham 2007). This phenomenon arises from particle-particle interactions and becomes significant when the volume fraction increases (Rusconi and Stone 2008). The resulting SID flux J_{SID}, is expressed in Eqs. S14 and S15 (Karthick and Sen 2017).

$$J_{SID} = -D_\gamma(V_f)\nabla V_f \qquad Eq.S14$$

where,

$$D_\gamma = \gamma a^2 \psi_\gamma(V_f) \qquad Eq.S15$$

with D_γ the SID coefficient, γ the shear rate and $\psi_\gamma(V_f)$ a factor which depends on the volume fraction but also on the shape and the deformability of particles.

The nominal shear rate in a microfluidic channel can be calculated with Eq. S16 (Gachelin et al. 2013; Liu et al. 2019).

$$\gamma = 6Q/wh^2 \qquad Eq.S16$$

with Q the flow rate, w the channel width and h the channel depth. The formula is correct when the velocity profile follows the Hagen-Poiseuille law, i.e., in confined and laminar flow.

A shear rate of 141 s^{-1} was calculated in a channel of 375×435 μm with flow of 100 mg/min.

Rusconi and Stone (2008) measured the SID coefficient as a function of the shear rate for plate-like clay particles with 1 μm radius at different volume fraction. They found a SID coefficient of about 6.6 at a shear rate of 141 s^{-1} for a 1% suspension. For comparison, the diffusion coefficient calculated with the Stokes-Einstein law results in 2.2 10^{-13}. The SID coefficient was higher for plate-like compared to spherical particles, highlighting the importance of anisotropy.
While clay particles have different properties compared to cells, this study provides a rough estimation of the importance of shape and the orders of magnitudes of D_φ and D_γ.

Given its suspected low contrast factor (due to low dimension), the elongated shape of *E. coli* could contribute to an increased SID, potentially impacting the efficiency of the acoustic process.

The shear rate is also responsible of increased heat transfer and change in suspension viscosity (Gadala-Maria and Acrivos 1980; Zydney and Colton 1988; Lopez and Graham 2007). Cell suspensions are indeed considered as non-Newtonian liquids (Gachelin et al. 2013; Liu et al. 2019). Gachelin et al. (2013) studied the viscosity of *E. coli* suspension at different solid contents as a function of the shear rate in a microfluidic chip. The curves showed a maximum viscosity at a shear rate of 20 s^{-1}, independent of the solid content. A shear rate of 141 s^{-1} was calculated in the previous section. This value drops to 64s^{-1} for a channel dimension of 375×250 µm and a flow rate of 15 mg/min. In our device, with *E. coli* suspension, the shear is therefore higher than the maximum of 20 s^{-1}. As a result, an increase in flow rate should decrease the viscosity. Based on the impact on T and on St, this means that an increase in the flow rate could result in an improvement in the acoustic processing capability. However, experimentally, it is not enough to counterbalance the rising of SID.

The comparison between the impact of solid content and shear rate on viscosity is an interesting exercise which could be part of a future development for each cell strain separately.

In any case however, the impact of the SID resulted in a maximum solid content for efficient acoustic processing with the current device geometry. For the applied experimental conditions, this limitation was defined for the yeast suspension between 5.30 10^8 and 2.65 10^8 cells/ml. The limit for the CHO suspension is above 4.5 10^6 cells/ml. The limit was not reached for the case of the tested *E. coli* suspension.

REFERENCES

Barnkob R, Augustsson P, Laurell T, Bruus H (2012) Acoustic radiation- and streaming-induced microparticle velocities determined by microparticle image velocimetry in an ultrasound symmetry plane. Phys Rev E - Stat Nonlinear, Soft Matter Phys 86:1–13. https://doi.org/10.1103/PhysRevE.86.056307

Chruickshank-Miller C (1924) The Stokes-Einstein law for diffusion in solution. 106:724. https://doi.org/https://doi.org/10.1098/rspa.1924.0100

Cussler EL (2007) Diffusion mass transfer in fluid systems, 3rd edn. Cambridge University Press, UK

Dhont JKG (1996) An introduction to dynamics of colloids, 2nd edn. Elsevier B.V., The nederlands

Gachelin J, Mino G, Berthet H, Lindner A, Rousselet A, Clement E (2013) Non-Newtonian viscosity of Escherichia coli suspensions. Phys Rev Lett 110:1–5. https://doi.org/10.1103/PhysRevLett.110.268103

Gadala-Maria F, Acrivos A (1980) Shear-induced structure in a concentrated suspension of solid spheres. J Rheol (N Y N Y) 24:799–814. https://doi.org/10.1122/1.549584

Karthick S, Sen AK (2016) Role of shear induced diffusion in acoustophoretic focusing of dense suspensions. Appl Phys Lett 109:1–5. https://doi.org/10.1063/1.4955274

Karthick S, Sen AK (2017) Improved understanding of the acoustophoretic focusing of dense suspensions in a microchannel. Phys Rev E 96:1–10. https://doi.org/10.1103/PhysRevE.96.052606

Krieger IM, Dougherty TJ (1959) A mechanism for non-Newtonian flow in suspensions of rigid spheres. Trans Soc Rheol 3:137–152. https://doi.org/10.1122/1.548848

Ladd AJC (1990) Hydrodynamic transport coefficients of random dispersions of hard spheres. J Chem Phys 93:3484–3494. https://doi.org/10.1063/1.458830

Ley MWH, Bruus H (2016) Continuum modeling of hydrodynamic particle-particle interactions in microfluidic high-concentration suspensions. Lab Chip 16:1178–1188. https://doi.org/10.1039/c6lc00150e

Liu Z, Zhang K, Cheng X (2019) Rheology of bacterial suspensions under confinement. Rheol Acta 58:439–451. https://doi.org/https://doi.org/10.1007/s00397-019-01155-x

Lopez M, Graham MD (2007) Shear-induced diffusion in dilute suspensions of spherical or nonspherical particles: Effects of irreversibility and symmetry breaking. Phys Fluids 19:1–10. https://doi.org/10.1063/1.2750525

Muller PB, Bruus H (2014) Numerical study of thermoviscous effects in ultrasound-induced acoustic streaming in microchannels. Phys Rev E - Stat Nonlinear, Soft Matter Phys 90. https://doi.org/10.1103/PhysRevE.90.043016

Rusconi R, Stone HA (2008) Shear-induced diffusion of platelike particles in microchannels. Phys Rev Lett 101:1–4. https://doi.org/10.1103/PhysRevLett.101.254502

Zydney AL, Colton CK (1988) Augmented solute transport in the shear flow of a concentrated suspension. PCH Physicochem Hydrodyn 10:77–96
